# Identifying the Primary Kinetic Factors Influencing the Anterior–Posterior Center of Mass Displacement in Barbell Squats: A Factor Regression Analysis

**DOI:** 10.3390/s25020572

**Published:** 2025-01-20

**Authors:** Diwei Chen, Dong Sun, Fengping Li, Dongxu Wang, Zhanyi Zhou, Zixiang Gao, Yaodong Gu

**Affiliations:** 1 Faculty of Sports Science, Ningbo University, Ningbo 315211, China; 2311040040@nbu.edu.cn (D.C.); 2211040065@nbu.edu.cn (F.L.); 2311040022@nbu.edu.cn (D.W.); 2212040169@nbu.edu.cn (Z.Z.); 2 Ningbo No. 2 Hospital, Ningbo 315010, China; 3 Faculty of Kinesiology, University of Calgary, Calgary, AB T2N 1N4, Canada; zixiang.gao@ucalgary.ca; 4 Faculty of Engineering, University of Szeged, H-6724 Szeged, Hungary

**Keywords:** barbell squat, Center of Mass (COM), joint moment, factor regression analysis

## Abstract

Background: Barbell squats are commonly used in strength training, but the anterior–posterior displacement of the Center of Mass (COM) may impair joint stability and increase injury risk. This study investigates the key factors influencing COM displacement during different squat modes.; Methods: This study recruited 15 male strength training enthusiasts, who performed 60% of their one-repetition maximum (1RM) in the Front Barbell Squat (FBS), High Bar Back Squat (HBBS), and Low Bar Back Squat (LBBS). Joint moments at both the hip, knee, and ankle were collected using a motion capture system and force plates, and a factor regression analysis was conducted using SPSS.; Results: In the FBS, primary factors influencing COM displacement included right knee adduction–abduction (38.59%), knee flexion–extension (31.08%), and hip internal–external rotation (29.83%). In the HBBS, they were right ankle internal–external rotation (19.13%), hip flexion–extension (−19.07%), and left knee flexion–extension (19.05%). In the LBBS, the key factors were left knee adduction–abduction (27.82%), right ankle internal–external rotation (27.59%), and left ankle internal–external rotation (26.12%).; Conclusion: The study identifies key factors affecting COM displacement across squat modes, with knee flexion–extension being dominant in the FBS and hip moments more significant in the HBBS and LBBS. These findings have implications for optimizing squat training and injury prevention strategies.

## 1. Introduction

As a foundational strength training exercise, the squat is widely utilized in sports such as athletics, volleyball, and weightlifting to effectively improve lower body strength and explosive power [1,2,3]. Squat training not only strengthens the thigh and glute muscles but also enhances dynamic movements like weightlifting, sprinting, and jumping, significantly boosting athletic performance [4,5].

The stable control of the Center of Mass (COM) is critical to the effectiveness and safety of squat training [6,7,8]. Different squat modes, such as the Front Barbell Squat (FBS), High Barbell Squat (HBBS), and Low Barbell Squat (LBBS), show distinct characteristics in COM displacement and joint load distribution. For instance, FBS demands a greater trunk control and places higher loads on knee flexion–extension, while HBBS and LBBS rely on the coordination of hip, knee, and ankle joints to maintain COM stability [9,10,11,12,13]. Abnormal COM displacement can cause joint instability, reduce training efficiency, and increase injury risk. This underscores the importance of optimizing COM control in squat training [14]. Dynamic changes in COM displacement are influenced by joint moments and trunk flexion angles, which vary across squat modes. For example, studies indicate that slower squats reduce the peak velocity of the COM and delay its occurrence, increasing movement difficulty and instability [15]. Additionally, an improper control of the anterior–posterior displacement of the COM can lead to uneven joint loading, elevate the risk of injury, and compromise the efficiency of force transfer during squats [16].

While existing studies provide valuable insights, they often fail to establish a clear quantitative relationship between COM displacement and joint moments, particularly in the sagittal plane. Despite extensive research on squat biomechanics, systematic and quantitative analyses of how anterior–posterior COM displacement affects torque distribution and movement stability remain limited [14,17,18,19,20,21,22]. To bridge this gap, this study employs factor regression analysis as a robust tool to explore the relationship between COM displacement and joint moments. Factor analysis reduces the complexity of interrelated biomechanical variables by consolidating them into fewer latent factors, while regression analysis quantifies each factor’s contribution to COM displacement [23,24]. This approach provides a systematic framework for identifying the key kinetic factors influencing torque distribution and stability across squat modes.

Based on the existing literature, we hypothesize that knee joint moments have the greatest influence on COM displacement in FBS, while hip and ankle joint moments play a larger role in HBBS and LBBS. By testing this hypothesis, this study aims to explore the relationships between these variables across different squat modes and identify the key dynamic factors influencing the anterior–posterior displacement of the COM in these squat modes. The findings will provide squat training guidance for athletes to enhance strength, optimize stability, and reduce the risk of injury, thereby offering empirical evidence for sports training and injury prevention.

## 2. Materials and Methods

### 2.1. Participants

The required sample size for this within-subject repeated measures design (one group × three conditions) was estimated using G*Power 3.1 (Franz Faul, Kiel, Germany) in accordance with Faul et al. [25]. Assuming a medium effect size (Cohen’s d = 0.5), an α level of 0.05, and a statistical power of 0.8, the calculation suggested a minimum of 9 participants. This estimation is consistent with previous research employing repeated measures ANOVA to assess the effects across different conditions [26].

A total of 15 male participants were recruited for this study (age: 20.2 ± 1.3 years, height: 169.3 ± 2.3 cm, and weight: 62.8 ± 3.6 kg), all of whom were strength training enthusiasts from Ningbo University with at least 2 years of formal strength training experience. All of the participants engaged in strength training at least three times per week, with each session lasting no less than one hour. None of the participants had undergone any lower limb or spinal surgeries in the past six months, nor had they been absent from physical activities due to injury for more than two weeks. The experimental protocol was approved by the Ethics Committee of Ningbo University (approval number: NO. RAGH20240820), and all participants provided written informed consent prior to participation.

### 2.2. Experimental Design

The study was conducted in the Biomechanics Laboratory of Ningbo University’s Research Academy of Grand Health, where kinematic and kinetic data were simultaneously collected using two force plates (Kistler, Winterthur, Switzerland) and a 12-camera Vicon motion capture system (Oxford Metrics Ltd., Oxford, UK). The data acquisition rates for kinematic and kinetic data were 200 Hz and 1000 Hz, respectively.

**Figure 1:**
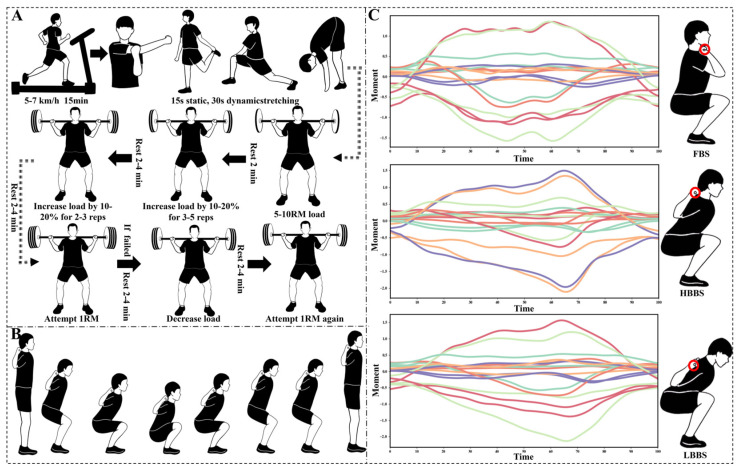
(**A**) The procedure for the 1RM test; (**B**) the entire motion phase of the squat test; (**C**) data of joint moments for the FBS, HBBS, and LBBS movements across three planes: sagittal, coronal, and transverse.

Before the formal testing, the 1RM for each participant was determined. Since different squat modes may result in varying 1RM values, the 1RM test was conducted every Sunday. The specific procedure was as follows:

Participants began by jogging on a treadmill at 5–7 km/h for 15 min to promote circulation and warm up the muscles. Following this, under the guidance of the experimenters, they performed a standardized light stretching routine to relax the muscles around the arms, thighs, calves, and ankles. This consisted of 15 s of static stretching and 30 s of dynamic stretching. Next, participants selected a weight that allows them to complete 5–10 repetitions comfortably and rested for 1 min after finishing. The weight was then increased by 10–20%, and they attempted 3–5 repetitions, followed by a 2 min rest. Subsequently, the weight was further increased by 0–20%, and participants performed 2–3 repetitions, resting for 2–4 min. Finally, participants added 2.5–5 kg to the load to begin the 1RM test. If they were unsuccessful, the weight was reduced appropriately after a 2–4 min rest and they attempted again (Figure 1A).

The 1RM test for FBS was conducted first, with the formal experiment starting on the third day after the test (i.e., Wednesday). The 1RM test for HBBS was then performed the following Sunday, and the formal experiment for HBBS began on the third day after the test (the following Wednesday). The 1RM test and formal experiment for LBBS followed the same procedure [27].

The formal testing procedure was as follows: After the warm-up, experimenters placed 39 markers on the participants’ bodies according to the Gait 2392 model in OpenSim, followed by static data collection. During the formal testing, the participant’s left foot was placed on Force Plate 1, and the right foot was placed on Force Plate 2. The force plates were synchronized with the motion capture system via a sync cable. The laboratory coordinate system was set as follows: the X-axis represented the anterior–posterior direction, with the positive direction being the one the participant faced; the Y-axis represented the left–right direction, with the positive direction aligned with the participant’s right leg; the Z-axis represented the vertical direction, with the positive direction pointing upward. The experimenters used a metronome to control the descent and ascent phases, each lasting 1.5 s, as the squat speed can influence joint moments. Upon receiving the start signal, the participant gradually bent their knees to lower the body at a constant speed until the surface of the thigh was below the knee joint. They then rose at a constant speed, ensuring that they could stand fully upright and lock the knee joint. No rebounding at the bottom of the squat or any downward motion during the ascent was allowed (Figure 1B).

To ensure both the safety of the experiment and the validity of the results, participants were required to perform the test with a load of 60% of their 1RM. During the test, their feet were positioned hip-width apart, with the toes externally rotated by 30°, and the test was conducted barefoot [28]. In this experiment, all participants were required to complete 4 valid trials. To prevent muscle fatigue, participants rested for 120 s after each squat.

### 2.3. Data Analysis

The raw motion capture data were initially pre-processed for necessary point deletion and interpolation using Nexus (version 2.10, Oxford Metrics Ltd., Oxford, UK). The processed data were then saved in C3D file format and imported into Visual 3D (version 6, C-Motion, Inc., Germantown, MD, USA) for further analysis. After creating the static model, it was then embedded into the C3D file, and a custom pipeline was used to calculate and output the COM displacement and the joint moments in the X, Y, and Z directions. All independent variable data were processed with a 4th-order low-pass filter, with cutoff frequencies set to 10 Hz for kinematic data and 20 Hz for kinetic data [29]. The filtered results were then carefully visually inspected. If a high-frequency amplitude noise is detected, the cutoff frequency was further reduced to eliminate the noise. Additionally, all variables were standardized based on the participants’ body weight. The independent variable data for all movement phases were normalized to 101 data points, aligning the data from different participants to the same time scale for an easier comparison (Figure 1C). The average values of all variables from the four squat trials for each participant were then used for subsequent analysis.

### 2.4. Statistical Analysis

Previous studies have extensively explored the modes of COM in the vertical and lateral directions [14]. However, the displacement of the COM in the anterior–posterior direction plays a crucial role in the balance and stability of the movement, and such modes may significantly affect training outcomes and athletic performance [12,19,30,31]. There-fore, this study focuses on the displacement of the COM in the anterior–posterior direction and employed IBM SPSS Statistics (version 26, IBM, Armonk, NY, USA) to conduct a stepwise multiple linear regression analysis to examine the correlation between the independent variables (joint moments of the hip, knee, and ankle in the X, Y, and Z axes) and the dependent variable (COM displacement in the X-axis direction) across all phases of the three squat modes. If the data did not meet the assumptions for regression analysis, dimensionality reduction would first be performed. The reduced factors would then be included alongside the dependent variable in the regression model for analysis. A significance level of *p* < 0.05 was used.

## 3. Results

### 3.1. Correlation Analysis Results Among Independent Variables

**Figure 2:**
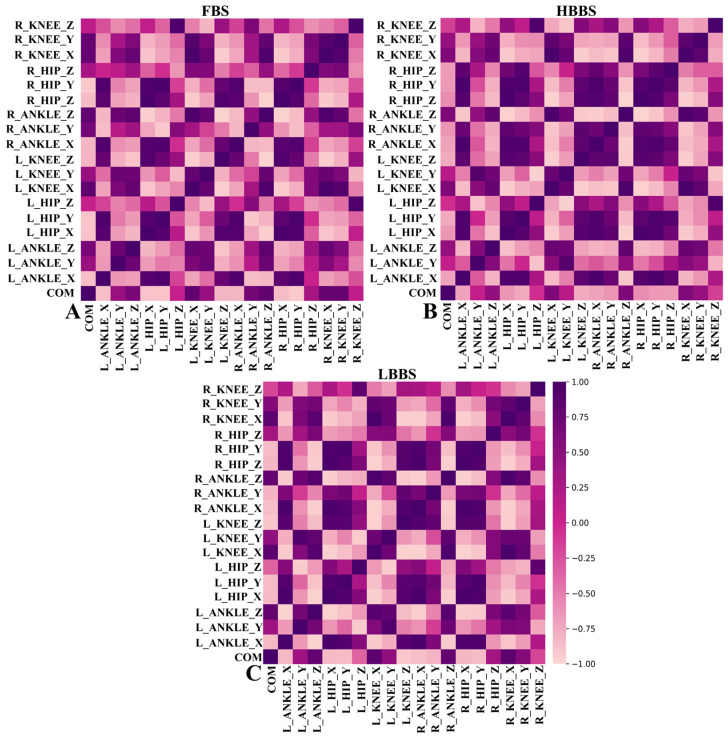
(**A**) Correlation between the joint moments of the hip, knee, and ankle in the X, Y, and Z axes and the COM during FBS; (**B**) Correlation between the joint moments of the hip, knee, and ankle in the X, Y, and Z axes and the COM during HBBS; (**C**) Correlation between the joint moments of the hip, knee, and ankle in the X, Y, and Z axes and the COM during LBBS.

As illustrated in Figure 2A, 78 pairs of strongly correlated variables were identified. Figure 2B presents 88 pairs of highly correlated variables, while the statistical analysis in Figure 2C reveals 91 pairs of strongly correlated variables. Moreover, the number of dark-colored blocks (indicating positive correlations) and light-colored blocks (indicating negative correlations) significantly exceeds that of magenta blocks (indicating no correlation). These results indicate a strong correlation between the independent variables in this study, which may lead to potential multicollinearity issues. Therefore, to avoid the interference of collinearity in the analysis results, direct regression analysis is not feasible. Instead, it is necessary to combine multiple independent variables into a smaller number of principal components to reduce dimensionality and effectively eliminate multicollinearity issues.

### 3.2. Results of Factor Analysis

The Kaiser–Meyer–Olkin (KMO) test results for the FBS, HBBS, and LBBS were 0.736, 0.762, and 0.781, respectively, indicating that the data are suitable for factor analysis. The KMO value ranges from 0 to 1, with values closer to 1 indicating stronger correlations between the variables. A KMO value greater than 0.6 is generally considered acceptable, which further supports the validity of the dimensionality reduction process in this study.

**Figure 3:**
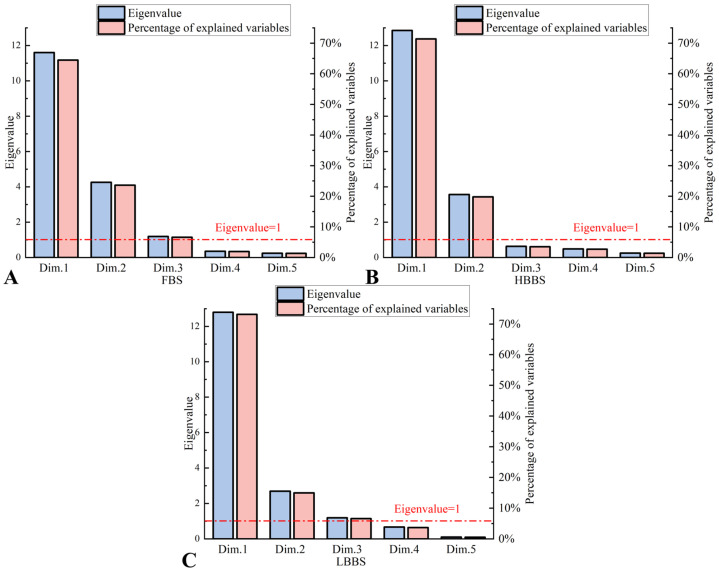
The left Y-axis shows the eigenvalues, while the right Y-axis indicates the contribution of the extracted principal components. (**A**) FBS; (**B**) HBBS; and (**C**) LBBS.

These results collectively confirm that the data are suitable for factor analysis, providing a solid foundation for the subsequent factor analysis. Based on the results of the factor analysis, only factors with eigenvalues greater than 1 were retained, and the maximum number of iterations was set to 25. As a result, three principal components were identified for both the FBS and LBBS, while two principal components were identified for the HBBS (Figure 3 and Table 1).

Based on Table 2, the expressions for the common factors in the FBS are as follows:

(1) $$PC1=98.1x_{1}-54.8x_{2}-67.7x_{3}\cdots \cdots \cdots -22.1x_{18}$$

(2) $$PC2=-5.3x_{1}+77.4x_{2}+53.8x_{3}\cdots \cdots \cdots -91.2x_{18}$$

(3) $$PC3=3.7x_{1}-20.1x_{2}+16.5x_{3}\cdots \cdots \cdots -29.9x_{18}$$

Based on Table 2, the expressions for the common factors in the HBBS are as follows:

(4) $$PC1=96.6x_{10}-4x_{2}-71.1x_{3}\cdots \cdots \cdots +13.1x_{18}$$

(5) $$PC2=-14.1x_{1}+89.7x_{2}+46.5x_{3}\cdots \cdots \cdots -95.4x_{18}$$

Based on Table 2, the expressions for the common factors in the LBBS are as follows:

(6) $$PC1=83.8x_{1}-21.5x_{2}-74.7x_{3}\cdots \cdots \cdots +5{.0x}_{18}$$

(7) $$PC2=-31.8x_{1}+86.7x_{2}+39.1x_{3}\cdots \cdots \cdots -94.4x_{18}$$

(8) $$PC3=-39.7x_{1}+23.2x_{2}+47.9x_{3}\cdots \cdots \cdots +26.3x_{18}$$

### 3.3. Results of Multiple Regression Analysis

**Figure 4:**
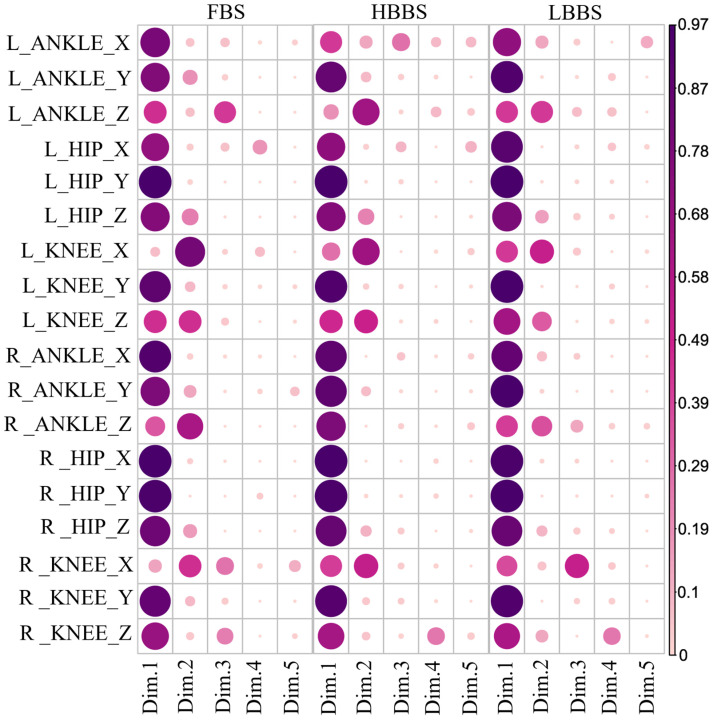
The higher the COS^2^ value, the greater the contribution of the variable to the principal component, thereby playing a more significant role in explaining the variance of the dependent variable.

After conducting multiple linear regression analysis on the dependent and independent variables extracted for each movement mode, we found that the model demonstrated significant advantages in both explanatory and predictive capabilities. The R^2^ values for the FBS, HBBS, and LBBS models were 0.884, 0.472, and 0.823, respectively. Additionally, the results of the analysis of variance revealed F-values of 245.504, 45.650, and 155.976, with significance levels well below 0.001, indicating that at least one independent variable has a statistically significant effect on the dependent variable. As shown in Figure 4, the COS^2^ contribution values for each variable under different movement modes indicate the importance of each original variable in the different principal components.

The statistical results clearly demonstrate that the multiple linear regression model developed in this study exhibits a high effectiveness and practicality in explaining and predicting COM displacement. The regression models for each condition are represented by Equations (9), (10), and (11), respectively.

(9) $$Y_{FBS}=-74PC1-3PC2-32.8PC3+1.366\times 10^{-13}$$

(10) $$Y_{HBBS}=-19.4PC1+2PC2-3.402\times 10^{-14}$$

(11) $$Y_{LBBS}=-24.7PC1+9.4PC2+3.6PC3+3.572\times 10^{-15}$$

Substituting Equations (1) to (3) into Equation (9) results in the following:

(12) $$Y_{FBS}=-15.66x_{1}-4.76x_{2}+13.83x_{3}-25.00x_{4}-23.49x_{5}-4.32x_{6}+29.13x_{7}+11.66x_{8}-22.11x_{9}\;-19.87x_{10}+14.84x_{11}+23.55x_{12}-24.56x_{13}-24.04x_{14}+29.83x_{15}+31.08x_{16}+38.59x_{17}\;+3.00x_{18}$$

Substituting Equations (4) to (5) into Equation (10) results in the following:

(13) $$Y_{HBBS}=-17.45x_{1}+9.69x_{2}+16.46x_{3}-18.98x_{4}-16.08x_{5}-11.66x_{6}+19.05x_{7}+14.47x_{8}-18.14x_{9}\;-17.76x_{10}-17.11x_{11}+19.13x_{12}-19.07x_{13}-17.46x_{14}-11.31x_{15}+18.83x_{16}+15.71x_{17}\;-11.77x_{18}$$

Substituting Equations (6) to (8) into Equation (11) results in the following:

(14) $$Y_{LBBS}=-25.05x_{1}+24.56x_{2}+26.11x_{3}-25.59x_{4}-19.38x_{5}-25.50x_{6}+25.76x_{7}+27.83x_{8}-21.86x_{9}\;-25.18x_{10}-10.30x_{11}+27.59x_{12}-25.94x_{13}-21.19x_{14}+19.51x_{15}+26.10x_{16}+25.29x_{17}\;-17.57x_{18}$$

Based on Equation (12), in the FBS, the three variables with the greatest influence on the anterior–posterior displacement of the COM are as follows: the abduction–adduction moment of the right knee (38.59%), the flexion–extension moment of the right knee (31.08%), and the internal–external rotation moment of the right hip (29.83%).

Based on Equation (13), in the HBBS, the three variables with the greatest influence on the anterior–posterior displacement of the COM are as follows: the internal–external rotation moment of the right ankle (19.13%), the flexion–extension moment of the right hip (−19.07%), and the flexion–extension moment of the left knee (19.05%).

Based on Equation (14), in the LBBS, the three variables with the greatest influence on the anterior–posterior displacement of the COM are as follows: the abduction–adduction moment of the left knee (27.82%), the internal–external rotation moment of the right ankle (27.59%), and the internal–external rotation moment of the left ankle (26.12%).

## 4. Discussion

In this study, we found that different squat movements not only involve changes in the joint moments but are also closely related to the position of the COM and body posture. In the FBS, the adduction–abduction moment and flexion–extension moment of the right knee, as well as the flexion–extension moment of the left knee, were positively correlated with the anterior–posterior displacement of the COM. This finding emphasizes the critical role of the knee joint in preventing excessive anterior displacement of the COM and supports our hypothesis that knee joint moments play a significant role in COM displacement. In the HBBS, the internal–external rotation moment of the right ankle and the flexion–extension moment of the left knee were positively correlated with the anterior–posterior displacement of the COM, while the flexion–extension moment of the right hip was negatively correlated. In the LBBS, the adduction–abduction moment of the left knee and the internal–external rotation moments of both ankles had the most significant influence on COM displacement, and all of them showed positive correlations.

This finding suggests that there is a certain degree of left–right moment asymmetry during the squat, which may stem from a preference for the dominant leg in daily activities, leading to a greater stability on one side. The difference in left–right stability may cause one side of the body to bear more load during the squat, particularly during the descending phase. Insufficient control on the weaker side may result in the anterior or posterior displacement of the COM. Since the COM is located at the center of the body’s line of gravity, any left–right imbalance can cause it to shift, thereby forcing the muscles to make additional adjustments to maintain balance. When the right side is stronger, the practitioner may tend to rely on the right hip, knee, and ankle joints to support the body. This could cause the COM to shift slightly toward the right, leading to instability in the anterior–posterior direction. This finding is consistent with previous studies, which reported that the adduction–abduction moment of the right knee influences the movement of the COM [32,33,34]. However, maintaining an imbalance in the left–right knee joint moments over an extended period may increase the risk of knee injury during the squat [35]. When the squat descends to its lowest point, insufficient support from the weaker side may lead to an excessive anterior displacement of the COM. Especially when there is an imbalance in the internal–external rotation and adduction–abduction moments at the hip and knee joints, the body’s center of gravity becomes unstable and moves excessively in the anterior–posterior direction, increasing the stress on the hip, knee, and ankle joints [36,37]. Therefore, to maintain COM stability, special attention should be given to unilateral loading exercises during training to balance left–right muscle strength and prevent excessive anterior–posterior fluctuations of the COM. By promoting balanced muscle development between the left and right legs, such as incorporating single-leg squats or lateral squats, the internal–externa stability and flexion–extension capacity of both knees can be effectively enhanced [35]. This, in turn, improves the overall symmetry of movement and COM control.

The internal–externa rotation moment of the right hip is regulated by key muscles, such as the gluteus minimus and piriformis, which help maintain the anterior–posterior balance of the COM by adjusting rotational forces. During the descent phase, these hip external rotators work synergistically to prevent excessive anterior COM displacement, maintaining hip stability and an upright spine [31,38,39]. In the ascent phase, hip internal rotators, such as the gracilis and adductor magnus, pull the COM back to the center, enhancing the overall squat stability. Similarly, the right knee abductor muscles, such as the vastus lateralis and tensor fasciae latae, play a critical role in maintaining knee joint stability and preventing excessive COM displacement during the descent phase, while knee adductors like the vastus medialis assist in maintaining a stable upward path during the ascent [40,41].To address muscle imbalances and improve COM stability, training should include unilateral exercises, such as single-leg squats and split squats, which enhance the coordination of hip external rotators and knee abductors [42,43]. Resistance band training focused on hip and knee adduction–abduction control can further reduce unnecessary anterior–posterior COM sway and improve movement fluidity. Strengthening the control of these moments ensures smooth and coordinated force generation, enhancing squat stability and reducing the risk of injury [44].

This study found that in the FBS, the dominant role of the knee joint is reflected in its support and weight-bearing functions. Since the FBS causes the COM to shift forward, the knee joint must maintain a large range of flexion–extension throughout the movement to effectively support the body [36,45]. The experimental results show significant variations in the flexion–extension angle and moment of the knee, further validating the central role of the knee joint in COM control. This is consistent with previous studies that identified the knee joint as the dominant factor in the FBS [46,47]. Additionally, the abduction and adduction moments of the knee are equally crucial for maintaining movement stability [39,48]. Particularly when a load increases, the knee abduction moment helps keep the knee in a neutral position, preventing the risk of knee valgus. Therefore, knee joint moment control should be a focus in squat training. At the same time, the internal–external rotation moment of the right hip also plays a significant role in COM control, particularly during the descent and ascent phases of the squat. This further demonstrates the active role of the hip joint in the squat, providing effective supportive assistance to the knee joint [32,49,50]. The hip joint primarily provides the necessary force support during the squat and helps adjust the COM position during knee flexion–extension [46,51]. The control of hip external and internal rotation is particularly crucial during significant knee flexion–extension, as it helps maintain a stable pelvic and trunk posture, preventing excessive anterior displacement or a posterior tilting of the COM [52]. In the HBBS, the internal–external rotation moment of the ankle dominates, indicating that compared to the FBS, the HBBS places higher demands on the ankle joint, further validating our hypothesis. In contrast to the control of the hip internal–external rotation moment, the flexion–extension moment of the hip has a more significant impact on the HBBS. This is because, in this mode, the COM is relatively higher, and in such a posture, the hip flexion–extension moment is crucial for body stability and COM control. The hip flexion–extension moment directly influences the anterior–posterior position of the COM by adjusting the hip joint angle. An effective flexion–extension moment helps keep the COM within an ideal range, reducing body instability. The HBBS movement itself emphasizes the integrated control of knee and hip flexion, meaning that the flexion–extension moment plays a central role in the entire kinetic chain. During the movement, the magnitude and effectiveness of the hip flexion–extension moment directly determine whether the athlete can successfully complete the squat and maintain stability, while the internal–external rotation moments play a supportive role in this process [2].

In practice, hip internal–external rotation control exercises, such as hip abduction and external rotation stretches, can be incorporated into squat training to enhance the support and stability of the hip joint. The ankle joint also has a greater range of motion in the FBS, particularly in terms of dorsiflexion. Ankle flexibility determines the overall stability of the lower limbs and affects the controllability of the anterior–posterior movement of the COM [53,54]. This study found that increasing the dorsiflexion angle of the ankle helps maintain body balance at the lowest point of the squat, preventing forward tipping. Previous studies have shown that in the HBBS, the dominant role of the hip joint is greater than that of the knee joint. Due to the barbell’s position behind the body, the hip’s range of motion is increased, making it a key point of force generation in the squat. The extension of the hip, in synergy with the gluteal muscles, ensures the posterior shift of the COM during the movement [48,49]. Additionally, the HBBS places high demands on the coordination of the hip, knee, and ankle joints. Under the dominant role of the hip joint, the knee and ankle joints must work in coordination to maintain body stability and balance. Compared to the HBBS, the dominant role of the knee joint is more pronounced in the FBS. The anterior placement of the barbell shifts the COM forward, causing the knee joint to bear more of the support and load-bearing responsibilities. As a result, the flexion–extension moment at the knee joint becomes a key factor in this movement. In this process, the hip and ankle joints work synergistically to control the COM [16,18]. Particularly at the lowest point of the squat, the posterior displacement of the hip joint and the dorsiflexion of the ankle joint play a crucial role in maintaining the stability of the body.

However, this study found that in the LBBS, the knee adduction–abduction moment of the left leg, the ankle internal–external rotation moment of the right leg, and the ankle internal–external rotation moment of the left leg has a significant impact on the COM displacement in the anterior–posterior direction. This result may be attributed to the fact that the participants primarily trained with the HBBS in their daily routine, leading to a weaker adaptation to the LBBS. As a result, larger moment differences were observed in the LBBS. Additionally, when transitioning from the HBBS to the LBBS in a short period, the knee joint is required to engage more to meet the dynamic demands of the deep squat position. This transition may result in an insufficient control of the knee joint in the LBBS, thereby increasing the influence of the knee flexion–extension moment. In the HBBS, due to the higher COM, the flexion–extension and internal–external rotation moments at the hip joint play a crucial role in maintaining stability [55]. In contrast, in the LBBS mode, as the COM lowers and the body inclination increases, the knee joint is required to take on a greater role in stability and support. In the HBBS, due to the higher center of gravity, the flexion–extension and internal–external rotation moments at the hip joint play a key role in maintaining stability [56]. In contrast, in the LBBS, as the center of gravity lowers and the body lean increases, the knee joint is required to assume more responsibility for stability and support.

Despite the differences among the three squat modes, the forward shift of the COM remains a common and significant characteristic. This phenomenon necessitates coordinated efforts across the hip, knee, and ankle joints to maintain body balance and effectively control the COM displacement in the anterior–posterior direction [57,58,59,60,61]. The coordination between the hip and knee joints in the squat demonstrates a strong interplay, with the internal–external rotation of the right hip joint working in conjunction with the flexion–extension, adduction, and abduction movements of the right knee to influence the COM displacement. This hip–knee interaction is crucial for maintaining balance during the squat, especially as the load increases. The active rotation of the hip joint provides more range of motion for the knee joint, thereby helping to alleviate the pressure on the knee. During the FBS, the ankle joint provides a supportive foundation for the entire kinetic chain, while the knee and hip joints work together to control the dynamic balance of the COM [58,59,60]. These three joints must maintain precise temporal coordination throughout the squat. For example, knee adduction and abduction must be coordinated with the internal–external rotation of the hip joint; otherwise, the movement may become unstable.

In practical training, static balance exercises, balance board training, and similar methods can be incorporated to improve the dynamic balance and coordinated control capabilities of the hip, knee, and ankle joints [37,52,53]. In both the HBBS and LBBS, the posterior movement of the hip joint helps prevent excessive forward displacement of the center of gravity. Additionally, its internal and external rotation capabilities are crucial for controlling the entire lower limb kinetic chain [57,61]. The flexibility and strength of the hip joint help reduce excessive pressure on the knee joint, effectively distributing the load and ensuring smooth execution of the movement. In the FBS, the dominant role of the knee joint is particularly prominent. Through forward flexion–extension movements, the knee joint helps maintain an upright torso, reducing the forward lean and further stabilizing the COM. The high activity level of the knee joint allows the lower limb to adapt to the anteriorly loaded position, aiding in the even distribution of the load. In the FBS, the forward displacement of the center of gravity requires the ankle joint to assume a greater dorsiflexion angle to counterbalance the forward lean of the upper body [36,46,55]. Therefore, the stability of the ankle joint and the grip strength of the foot are crucial for maintaining COM balance. Insufficient ankle flexibility may lead to instability of the COM, thereby affecting the overall stability of the movement. Due to the high reliance on knee flexion–extension in the FBS, this mode is particularly suitable for individuals aiming to strengthen knee flexion–extension strength and enhance the quadriceps and anterior chain musculature [36]. Additionally, the FBS requires maintaining an upright torso and preventing a forward lean, which helps improve the control of the core region. This is especially beneficial for sports that require core stability, such as weightlifting and athletics. In contrast, the FBS has relatively lower flexibility requirements for the hip and ankle joints, making it more suitable for individuals with a lower flexibility [35,53]. At the same time, the FBS places higher demands on balance and coordination, helping to improve the overall movement control abilities of the practitioner. The LBBS not only emphasizes the role of the hip joint but also specifically focuses on the knee flexion–extension moment [41]. Especially in deeper squat positions, the knee joint is required to bear more load and assume a greater responsibility for stability. The LBBS places higher flexibility demands on the hip and ankle joints. Practitioners must ensure sufficient flexibility in these joints to prevent injuries during the movement [14,18,46]. The HBBS demonstrates a relatively neutral characteristic, not favoring either the anterior or posterior chain excessively. It integrates the core strength focus of the FBS, which aids in maintaining posture, while also addressing the flexibility and force output demands of the LBBS. Consequently, the HBBS provides a balanced training approach, making it well–suited for individuals seeking to develop multiple physical attributes within their squat technique.

Despite providing valuable insights, this study has several limitations. Factor regression analysis simplifies complex biomechanical interactions, potentially overlooking subtle yet important factors, which limits a comprehensive understanding of the underlying mechanisms. Additionally, the factor analysis process may obscure the specific contributions of individual variables. The use of a single-gender sample and low data diversity further restricts the generalizability and robustness of the findings. Lastly, this study primarily focused on the overall influence of joint moments on COM displacement, without examining the dynamic changes during different squat phases (descent, hold, and ascent) or considering kinematic parameters such as joint angles and angular velocities. Future research should employ more flexible nonlinear methods, increase sample diversity, and analyze squat phases separately to provide a more comprehensive understanding of the mechanisms influencing COM displacement.

## 5. Conclusions

Through the analysis of lower limb joint moments in each mode, we found that in the FBS, the knee flexion–extension moment plays a dominant role in the anterior–posterior displacement of the COM, whereas in the HBBS and LBBS, the internal–external rotation and flexion–extension moments at the hip joint are crucial for COM control. These findings provide theoretical support for optimizing squat training and offer valuable guidance for practitioners seeking to enhance performance and reduce the risk of injury. Future research should explore the influence of additional biomechanical factors on COM displacement, assess the applicability of these findings across diverse populations, and investigate the effects of varying training intensities or speeds on the identified key factors. Incorporating such directions will help deepen our understanding of lower-limb kinetics and enhance the practical value of squat training recommendations.

## Figures and Tables

**Table 1:** Extracted Principal Component Factors.

Squat Mode	Component	Total	Percentage of Variance	Cumulative %
FBS	1	11.600	64.446	64.446
FBS	2	4.252	23.624	88.069
FBS	3	1.187	6.596	94.665
HBBS	1	12.851	71.396	71.396
HBBS	2	3.562	19.788	91.184
LBBS	1	13.173	73.184	73.184
LBBS	2	2.692	14.954	88.139
LBBS	3	1.186	6.589	94.728

**Table 2:** Factor Analysis Rotated Component Matrix.

Squat Mode	Variable	Factor 1	Factor 2	Factor 3	Variable	Factor 1	Factor 2	Factor 3
FBS	L_ANKLE_X	0.981	−0.053	0.037	R_ANKLE_X	0.949	−0.056	−0.087
FBS	L_ANKLE_Y	−0.548	0.774	−0.201	R_ANKLE_Y	−0.874	−0.429	−0.117
FBS	L_ANKLE_Z	−0.677	0.538	0.165	R_ANKLE_Z	−0.800	0.483	0.347
FBS	L_HIP_X	0.932	−0.230	−0.266	R_HIP_X	0.868	−0.342	−0.310
FBS	L_HIP_Y	0.984	0.113	−0.126	R_HIP_Y	0.982	0.019	−0.163
FBS	L_HIP_Z	−0.165	−0.860	−0.381	R_HIP_Z	0.032	0.342	0.859
FBS	L_KNEE_X	−0.677	0.472	0.544	R_KNEE_X	−0.671	0.432	0.588
FBS	L_KNEE_Y	−0.306	0.852	0.351	R_KNEE_Y	−0.568	0.175	0.771
FBS	L_KNEE_Z	0.779	−0.499	−0.323	R_KNEE_Z	−0.221	−0.912	−0.299
HBBS	L_ANKLE_X	0.966	−0.141	/	R_ANKLE_X	0.967	−0.170	/
HBBS	L_ANKLE_Y	−0.040	0.897	/	R_ANKLE_Y	0.757	−0.453	/
HBBS	L_ANKLE_Z	−0.711	0.465	/	R_ANKLE_Z	−0.856	0.490	/
HBBS	L_HIP_X	0.926	−0.360	/	R_HIP_X	0.883	−0.441	/
HBBS	L_HIP_Y	0.991	0.036	/	R_HIP_Y	0.979	−0.119	/
HBBS	L_HIP_Z	0.116	−0.969	/	R_HIP_Z	0.908	0.373	/
HBBS	L_KNEE_X	−0.790	0.591	/	R_KNEE_X	−0.754	0.629	/
HBBS	L_KNEE_Y	−0.311	0.927	/	R_KNEE_Y	−0.606	0.562	/
HBBS	L_KNEE_Z	0.844	−0.411	/	R_KNEE_Z	0.131	−0.954	/
LBBS	L_ANKLE_X	0.838	−0.318	−0.397	R_ANKLE_X	0.879	−0.339	−0.314
LBBS	L_ANKLE_Y	−0.215	0.867	0.232	R_ANKLE_Y	0.905	0.010	0.345
LBBS	L_ANKLE_Z	−0.747	0.391	0.479	R_ANKLE_Z	−0.770	0.587	0.227
LBBS	L_HIP_X	0.849	−0.336	−0.395	R_HIP_X	0.850	−0.388	−0.331
LBBS	L_HIP_Y	0.928	−0.008	−0.349	R_HIP_Y	0.916	−0.086	−0.379
LBBS	L_HIP_Z	0.180	−0.948	−0.214	R_HIP_Z	−0.307	0.210	0.842
LBBS	L_KNEE_X	−0.860	0.446	0.202	R_KNEE_X	−0.822	0.533	0.128
LBBS	L_KNEE_Y	−0.361	0.812	0.403	R_KNEE_Y	−0.445	0.692	0.289
LBBS	L_KNEE_Z	0.950	−0.286	−0.037	R_KNEE_Z	0.050	−0.944	0.263

## Data Availability

The data that support the findings of this study are available on reasonable request from the corresponding author.
